# Evaluation of polymorphism of *P53* protein codon 72 in oral lichen planus by PCR technique

**DOI:** 10.15171/joddd.2018.038

**Published:** 2018-12-19

**Authors:** Seyed Hosein Tabatabaei, Mohammad Hasan Sheikhha, Mahammad Hasan Akhavan Karbasi, sedighe zarmehi, Mehdi Hoseini

**Affiliations:** ^1^Department of Oral and Maxillofacial Pathology, Member of Social Determinants of Oral Health Research Center, Shahid Sadoughi University of Medical Sciences, Yazd, Iran; ^2^Department of Genetics, Research and Clinical Center for Infertility, Shahid Sadoughi Medical Sciences University, Yazd, Iran; ^3^Department of Oral Medicine, Faculty of Dentistry, Shahid Sadoughi University of Medical Sciences, Yazd, Iran; ^4^Department of Oral and Maxillofacial Pathology, Faculty of Dentistry, Rafsanjan University of Medical Sciences, Rafsanjan, Iran

**Keywords:** codon 72, malignant transformation, oral lichen planus, *p53* polymorphism

## Abstract

***Background.*** Our research was aimed to study *p53* protein codon 72 polymorphism, a single base pair change of either arginine (Arg; CGC) or proline (Pro; CCC) that creates 3 distinct genotypes in reticular oral lichen planus (OLP) in comparison to oral SCC which is the most common oral mucosal malignancy as positive control and inflammatory fibrous hyperplasia (IFH) lesion as negative control.

***Methods.*** Seventy paraffin-embedded tissue samples (30 OLP, 20 OSCC and 20 IFH) were studied. DNA was purified and amplified using allele-specific polymerase chain reaction to detect polymorphism. The final amplified products were identified using gel electrophoresis. Data were analyzed using chi-squared test and odds ratio.

***Results.*** The mean ages in the OLP, OSCC and IFH groups were 43.28, 58.2 and 53.47 years, respectively, with significant differences. There were no significant differences in gender between the three groups (P=0.413); however, the differences in genotypes and alleles were significant between the three groups (P=0.021 and P=0.030, respectively). By considering IFH as a reference, the frequency of proline allele in OLP and OSCC was significantly higher than that of arginine allele (P=0.015 and P=0.028, respectively). In addition, by considering OSCC as a reference and at P=1, there were no significant differences in the frequencies of alleles between OSCC and OLP.

***Conclusion.*** The results might indicate the premalignant potential of OLP, and such polymorphism might be a genetic predisposing factor for conversion of OLP to OSCC. In addition, in the subjects evaluated the proline allele was considered a risk factor.

## Introduction

Lichen planus is a relatively common chronic mucocutaneous lesion which usually affects the oral mucosa, with a prevalence rate of 0.1‒2.2%.^1^ The condition is usually manifested at middle ages and rarely affects children.^2^ The oral lesions are generally in reticular or erosive forms.^1^ Despite widespread research, the etiology of the condition is unknown, although an autoimmune etiologic agent has been suggested.^3^ Although the WHO has classified lichen planus as a premalignant condition,^4^ there is controversy over the premalignant nature of these lesions and the hypothesis on the possibility of progression of oral lichen planus (OLP) to oral squamous cell carcinoma (OSCC) dates back to 1910.^5^ Malignant changes have been reported in 2‒3% of patients with OLP.^6^ However, many cases reported as malignant changes lack hard evidence. Some reported cases might not have been true lichen planus cases and might have in fact been dysplastic leukoplakia in association with a secondary inflammatory lichenoid infiltration.

In addition, it has been reported that since SCC and lichen planus are not rare conditions, they might occur in one individual simultaneously, with no relationship between them. Furthermore, some researchers believe that the atrophic epithelium of the erosive form of lichen planus has a higher susceptibility to carcinogens, increasing the risk for malignant changes in these lesions.^1^

*P53* gene, as the most important tumor suppressor gene, is located on the short arm of chromosome 17 (17p13.1) and has 11 exons.^7^ This gene encodes a nuclear phosphoprotein with 393 amino acids, the natural function of which is to protect the genome against injuries. This process results in the repair of genome or apoptosis, leading to the elimination of carcinogenic cells.^8^ *p53* has an important role in the preservation of the integrity of genome and usually undergoes mutation in human carcinoma or becomes defective in relation to its function. In normal cells, *p53* is destroyed immediately and is not detected by immunohistochemical (IHC) staining.^5^ Mutation of *p53* gene might result in the formation of a defective protein with greater stability and higher durability in the tissue, which is the basis for the use of IHC to trace mutated *p53* products.^9,10^

Polymorphism of the codon 72 on exon 4 of the gene *p53* is the first and most commonly known polymorphism of this gene. In this polymorphism arginine (CGC) is converted to proline (CCC) in the protein sequence. It has been suggested that two alleles of the codon 72 have different carcinogenic properties.^11^ In this context, the proline allele is associated with an increased risk for the SCC of the head and neck region.^12^

Mutation of *p53* gene is the most important genetic change in human cancers^13^ and some studies have shown some degrees of mutation in *p53* gene in OLP patients, especially in women.^5^ In addition, some studies have shown apoptosis in lichen planus due to the expression of *p53* protein.^14^ On the other hand, another study compared the molecular characteristics of lichen planus and dysplastic and reactive lesions and normal oral mucosa and did not fully support the premalignant nature of oral lichen planus.^1^

Considering the contradictory results in this respect^1,5^ and since the high expression of *p53* during IHC evaluations might have no direct relationship with the mutation of the genome, evaluation of mutations in *p53* gene by molecular-level analyses are useful with the use of PCR technique. This technique has 95% sensitivity for tracing point mutations in *p53* gene.^15^

The present study was undertaken to evaluate the most common mutation of codon 72 of *p53* protein in reticular oral lichen planus in comparison to oral SCC as an oral mucosa malignant lesion, in which the mutation of the tumor suppressor gene of *p53* has been commonly identified.^1^ An inflammatory and hyperplastic benign lesion such as an epulis fissuratum was used as a negative control. On the other hand, all the lichen planus samples were selected from the reticular type and no erosive forms were included in the study in order to decrease the possible effect of carcinogenic agents through the atrophic epithelium or ulcerated erosive lichen planus as a confounding factor.

Despite a few studies on premalignant potential of OLP in the world, there are very limited data available based on molecular analysis and in Iran no tissue sample mutational analysis has been carried out in this context. Therefore, in the present study we aimed to investigate the premalignant potential of OLP by using PCR technique on tissue samples in Yazd Province in the center of Iran, with several patients referring from other cities and neighboring provinces.

## Methods

In this retrospective case‒control study, the case samples were selected, using the census method, from the fixed and paraffin-embedded samples in the Archives of the Department of Pathology, Yazd Faculty of Dentistry in the Central Iran, covering a period from 2001 to 2014. The samples had a definite diagnosis of reticular lichen planus, with adequate tissue measuring at least 5×5 mm.

The control samples consisted of two groups of OSCC and inflammatory fibrous hyperplasia (IFH) without any cellular dysplasia, in the same Archives.

Our research was aimed to study *p53* codon 72 polymorphisms, a single base pair change of either arginine (Arg; CGC) or proline (Pro; CCC) that creates 3 distinct genotypes: homozygote for Arg (Arg/Arg), homozygote for Pro (Pro/Pro) and heterozygote (Pro/Arg). In this regard, the 10% formalin-fixed and paraffin-embedded specimens were serially sectioned by a microtome. The microtome blade was disposable and all tools and the surrounding area were cleaned with xylene and ethanol after processing each sample to avoid contamination among samples. The first and last sections were stained with hematoxylin-eosin (H&E) to confirm the diagnosis. Paraffined tissue was dissolved with xylene; and genomic DNA was extracted by means of Biospin FFPE Tissue Genomic DNA Extraction Kit.

To investigate the variant of each sample, genomic DNA was amplified by allele-specific polymerase chain reaction. Two sets of primers (forward [F] and reverse [R]) were used to amplify the Arg and Pro alleles as follows:

Arg F: TCC CCC TTG CCG TCC CAA

Arg R: CTG GTG CAG GGG CCA CGC

Pro F: GCC AGA GGT TGC TCC CCC

Pro R: CGT GCA AGT CAC AGA CTT

On the basis of which set of primers could attach, the polymerase chain reaction product was 177 base pairs (bp) for the Pro allele and 135 bp for the Arg allele. To achieve these products, 50 ng of genomic DNA extracted from each sample was added to a tube with a total volume of 25 μL, containing Taq DNA Pol 2X Master Mix Red (Ampliqon) with 1.5 mmol/L of MgCl_2_ and 2-U Taq polymerase, and 10 pmol/μL of each primer.

Amplification was performed in a touch-down manner, including the initial denaturation process at 94°C for 5 minutes followed by 10 cycles each consisting of denaturation at 94°C for 30 seconds and annealing at 68−62°C, then for the remaining 25 cycles, annealing temperatures were 62−58°C, with extension at 72°C for 30 seconds in each cycle.

The final amplified products were identified using gel electrophoresis (2% agarose gel with DNA safe stain).

After PCR amplification, data were collected and entered into and were analyzed with chi-squared test and odds ratio using SPSS 18.

## Results

A total of 70 paraffin blocks, consisting of 30 OLP, 20 OSCC and 20 IFH samples, were evaluated in the present study. During extraction of DNA and PCR procedures, two OLP samples and one IFH sample were excluded from the study because they did not exhibit acceptable bands.

The mean ages in the OLP, OSCC and IFH groups were 43.28, 58.2 and 53.47 years, respectively. ANOVA revealed significant differences in the mean ages between the three groups (P=0.04).

Table 1 presents the frequencies of polymorphism of gene *p53* codon 72 in the three groups in terms of gender, genotype and allele. Based on data presented in the Table, there were no significant differences in gender between the three groups (P=0.413); however, the differences in genotypes and alleles were significant between the three groups (P=0.021 and P=0.030, respectively). Table 2 presents the frequencies of alleles in patients with OLP and OSCC compared to those with IFH and also the frequencies of alleles in patients with OLP compared to those with OSCC.

**Table 1:** Frequencies of polymorphism of gene *p53* codon 72 in patients in terms of gender, genotype and allele

**Patients**		**OLP, N (%)**	**OSCC, N (%)**	**IFH, N (%)**	**P-value**
**Variables**					
**Gender**	**Male**	4 (21.1)	11 (39.3)	7 (35.0)	0.413
	**Female**	15 (78.9)	17 (60.7)	13 (65.0)	
**Genotype**	**Arg/Arg**	12 (63.2)	6 (21.4)	4 (20.5)	0.021
	**Arg/Pro**	7 (36.8)	20 (71.4)	15 (75.0)	
	**Pro/Pro**	0 (0.0)	2 (7.1)	1 (5.0)	
**Allele**	**Arg**	31 (81.6)	32 (57.1)	23 (57.5)	0.030
	**Pro**	7 (18.4)	24 (42.9)	17 (42.5)	

Chi-squared test OLP: oral lichen planus, OSCC: oral squamous cell carcinoma, IFH: inflammatory fibrous hyperplasia

Based on data presented in Table 2 and at a confidence interval of 95% and by considering IFH as a reference, the frequency of proline allele in OLP and OSCC was significantly higher than that of arginine allele (P=0.015 and P=0.028, respectively).

**Table 2:** Frequencies of alleles in patients compared to references

**Patients**	**Allele**		**Total**	**P-value**	** CI)**
	**Arg, N (%)**	**Pro, N (%)**			
IFH	31 (81.6)	7 (18.4)	38 (100.0)	-	Ref
OLP	32 (57.1)	24 (42.9)	56 (100.0)	0.015	3.321
OSCC	54 (69.2)	24 (30.8)	78 (100.0)	0.028	3.273
OSCC	23 (57.5)	17 (42.5)	40 (100.0)	-	Ref
OLP	32 (57.1)	24 (42.9)	56 (100.0)	1.0	0.986

Fisher’s exact test OR: odd ratio, CI: confidence interval, Ref: reference

In addition, by considering OSCC as a reference and at P=1, there were no significant differences in the frequencies of alleles between OSCC and OLP.

## Discussion

The controversy over the potential of OLP to convert to malignancy dates back to 1910 and many studies have been carried out to date on the subject.^16^ The malignant potential of OLP has been reported to be 0‒10% in different studies in patients after 1.5‒10-year follow-ups, depending on the subjects’ characteristics and study design.^17^

One of the strong points of the present study was exclusion of erosive lichen planus. In a study by Yanatatasaneeji et al^14^ in Thailand such a problem was resolved by evaluating lichen planus cases in two separate erosive and non-erosive groups.

There is a band of inflammatory cell infiltration in the histopathological view of OLP; therefore, it is logical to select a condition in the control group, in which inflammation is one of the principal characteristics. As a result, inflammatory fibrous hyperplasia (IFH) was selected in this study to evaluate the effect of inflammation on the polymorphisms of *p53* gene. Our search brought up only one study in which the immunohistochemical expression of *p53* gene has been compared in OLP and IFH.^18^ Use of PCR instead of IHC might be considered one of the advantages of this study in order to evaluate the effect of inflammation.

It has been demonstrated that the presence of proline (Pro) allele or Pro/Pro genotype in the *p53* protein is a potential risk factor for malignancies of the lungs, esophagus, abdomen, breasts, nasopharynx, prostate, liver and colon; in other populations, the arginine allele (Arg) or Arg/Arg genotype in the *p53* protein is a risk factor for malignancies of uterus, colon, breasts and pharynx.^19^ In the present study, too, since the frequencies of Pro allele and Arg/Pro and Pro/Pro genotypes were significantly higher in the SCC group compared to the benign IFH group, proline allele was considered a risk factor. In a study by Sullivan et al,^12^ too, proline allele was considered a risk factor for SCC of the head and neck region.

The results of the present study showed a relationship between polymorphism of codon 72 of *p53* gene and lichen planus, similar to the relationship between polymorphism of codon 72 of *p53* gene in OSCC; in this context, the frequencies of alleles in OLP patients were not significantly different from those in OSC patients (P=1). In addition, in both OLP and OSCC groups polymorphism was higher than that in IFH group, with proline allele in OLP and OSCC being significantly more frequent than arginine allele (P=0.015 for OLP and P=0.025 for OSCC).

The results of the present study are consistent with those of Yanatatsaneeji et al,^14^ who considered lichen planus a precancerous lesion; however, in the present study, the heterozygous genotype (Arg/Pro) exhibited the highest frequency, with 71.4% in lichen planus and 75% in SCC, which is contrary to the results Yanatatsaneeji et al study, in which Pro/Pro genotype had the highest frequency. In both studies, there was a significant relationship between the frequencies of polymorphism of codon 72 and the allele, with a P-value of 0.030 in the present study and a P-value of 0.001 in the study above.

In another case‒control study, Ogmandottir et al^5^ in Iceland reported that a high rate of mutation in OLP is an indication of the premalignant nature of this lesion.

On the contrary, in a study by Ghapanchi et al^20^ no significant differences were found in the polymorphism of codon 72 of *p53* gene between the OLP and control group, which included blood samples from healthy subjects, neither in the genotype (P=0.3) nor in the allele (P=0.5). The advantage of the present study over the study above was the fact that there were two positive and control groups in the present study, which made it possible to compare OLP simultaneously with OSCC and IFH.

The results of a study by Schifter et al^15^ in Australia do not coincide with those of the present study. After IHC evaluation of lichen planus, they evaluated mutations in 10 OLP cases with the highest staining using the PCR technique and did not report any mutations. Mutation frequency in the study above was less than that expected.

Such discrepancies in the results might be attributed to techniques used to take samples from tissues to extract DNA. In the present study, attempts were made to use paraffin blocks with adequate tissue to minimize the odds of false negative results. In addition, adequate care was exercised during DNA extraction procedures and a new scalpel blade was used to cut each block to minimize the odds of false positive results.

Several other studies have been undertaken to evaluate the malignant potential of OLP by evaluation of *p53* mutations with the use of IHC. However, it should be pointed out that in IHC evaluation, excessive expression of *p53* protein might be due to the presence of inflammatory mediators in the connective tissue similar to the situation in oral lichen planus, without any direct relationship with the mutation in the genome, which might be considered one of the disadvantages of IHC technique for the evaluation of mutations.

De Sousa et al^21^ did not report any significant differences in the expression of *p53* between OLP and OSCC (P>0.05), consistent with the results of the present study. They believed that such a result was an indication of the malignant potential of OLP.

Fernando et al^22^ reported the similar expression of *p53* protein in OLP and epithelial dysplasia (P=1), consistent with the results of the present study, which might be an indication of the malignant potential of both lesions.

Safadi et al^23^ reported a significantly higher expression of *p53* in OLP compared to that in oral mucositis, normal oral mucosa and keratosis (P<0.001) and a lower expression compared to OSCC (P<0.001), consistent with the results of the present study. However, no significant differences were detected from mild dysplasia (P=0.85). They concluded that OLP should be accurately followed to prevent malignant transformation.

The results of an IHC study by Lee et al^24^ do not coincide with the results of the present study. They failed to show a premalignant nature for OLP due to the low expression of *p53* compared to SCC (P<0.001). One of the strong points of that study was evaluation of age, gender, the duration of the lesion, the location and size of the lesion, the number of locations involved, presence of pain, local stimuli and use of drugs and alcohol. They did not find any relationships between variables above and expression of *p53* in OLP (P>0.05). However, the condition was more severe in drug users and in erosive and atrophic forms of OLP.

The discrepancies between the results of different studies might be attributed to differences in laboratory techniques used to determine *p53* sequence or the IHC technique and also ethnic variations, geographic locations and nutritional habits in different regions.

In the present study, *p53* codon 72 polymorphism did not exhibit significant differences in terms of gender (P=0.913). In this context, there were 7 males and 13 females in the OSCC group, contrary to the findings of Neville et al,^1^ in which the overall male-to-female ratio was 3:1 in the United States. Such discrepancy might be explained by the small sample size in the OSCC group in the present study. Lichen planus was more frequent in females in the present study with 17 females compared to 11 males. Based on the reports made by Neville, too, a female-to-male ratio for lichen planus was 3:2. In a study by Lee, too, the mutation was not significant in terms of gender (P>0.05).

There were significant differences in mean ages between the three groups in the present study (P=0.04). The mean ages in the OLP and OSCC patients were 43.28 and 58.20 years, respectively, indicating that SCC occurred at an older age, contrary to the results of a study by Lee, in which mutation had no significant relationship with age.^24^

Based on the results of a study by Qian et al,^18^ long-term and chronic inflammation results in a special environment under the subepithelial stroma, leading to mutation of oncogenes and promotion of the proliferation of adjacent epithelial cells. Based on this hypothesis, subepithelial inflammation might result in the proliferation of epithelial cells, leading to malignant transformation.^25^ This hypothesis might explain the presence of Pro risk allele in a small number of IFH cases (18.4%), which is an inflammatory and hyperplastic lesion. However, it should be pointed out that the lesion should persist for a long time to undergo malignant changes because it has been demonstrated that in the short term the natural protective mechanisms of cells, including inflammations, are activated against carcinogenic agents.^18^ Some researchers, including Gnepp,^26^ believe that in OLP, malignancies develop from lesions that have persisted for a long time.

Since no malignancies were observed at the time lichen planus was diagnosed in the present study and since after taking a biopsy and fixing and embedding it in paraffin no cellular and molecular changes were possible in the samples, it is improbable for inflammation to be a precancerous condition in lichen planus as mentioned above. In addition, if inflammation is to be considered a carcinogenic agent, in IFH lesions the *p53* codon 72 polymorphism should have been common. Therefore, inflammation might slightly increase proline risk allele but it alone does not result in cancer in lesions that have recently developed.

In this context, in a study by Oliveria et al,^18^ too, IHC expression of *p53* was positive and significant in OLP and OSCC and poor or absent in IHF. There was no significant relationship between OLP and OSCC (P>0.05). However, there was a significant relationship between OLP and IHF (P<0.05). The results above are consistent with those of the present study. They attributed the poor expression of *p53* protein to the chronic and diffuse infiltration of inflammatory cells in IHF, which results in changes in the basal cells in response to stressful situations such as oxidative injuries, finally leading to *p53* aggregation.

In another study, expression of *p53* protein was evaluated in aphthous stomatitis, IFH and normal mucosa and significant differences were observed between IFH and normal mucosa (P<0.05), which was attributed to inflammation.^27^

In general, a hypothesis that can be presented in relation to the premalignant nature of OLP should be sought out in the inherent and histopathological characteristics of lichen planus and in the biologic activity of the two alleles of *p53* gene. Based on a study by Pim et al,^11^ proline allele has lower potential to induce apoptosis compared to arginine. Since degeneration of keratinocytes in lichen planus is a result of apoptosis, despite polymorphism of arginine compared to protein, less apoptosis takes place, which paves the way for excessive proliferation and malignant transformation of lichen planus. Based on a study by Bonafe et al,^28^ too, polymorphism of codon 72 of *p53* gene is related to apoptosis. In a study by Yanatatsaneeji et al,^14^ too, apoptosis, in which *p53* has a role, was considered to have a role in the pathogenesis of lichen planus.

## Conclusion

Considering the significance of the frequency of polymorphism of codon 72 of *p53* gene in the three groups studied in terms of genotype and allele, the results of the present study might indicate the premalignant potential of OLP, and such polymorphism might be a genetic predisposing factor for conversion of OLP to OSCC. In addition, in the subjects evaluated the proline allele was considered a risk factor. Therefore, patients with OLP lesions require accurate and constant follow-ups to prevent malignant transformations and diagnose and treat even minor changes in the oral lesions at early stages.

## Acknowledgment

We sincerely appreciate Mrs. Hoseini for her cooperation to gain access to pathology records and Dr. Fesahat and Mrs. Mortezaee for their technical assistance.

## Authors’ contributions

Tabatabaei SH, Sheikhha MH, Zarmehi S were involved in designing the study and collection of data. All the au-thors were involved in drafting and final approval of the manuscript.

## Funding

The study was financially supported by Shahid Sadoughi university of medical sciences, Yazd, Iran.

## Competing interests

We have no conflict of interest to declare.

## Ethics approval

The ethical clearance was obtained from the institutional ethics committee. All the patients involved in the study were consent to use their paraffin-embedded tissue sam-ples.
